# Avoidance of an aposematically coloured butterfly by wild birds in a tropical forest

**DOI:** 10.1111/een.12335

**Published:** 2016-06-25

**Authors:** DENISE D. DELL'AGLIO, MARTIN STEVENS, CHRIS D. JIGGINS

**Affiliations:** ^1^ Butterfly Genetics Group, Department of ZoologyUniversity of CambridgeCambridgeU.K.; ^2^ Smithsonian Tropical Research InstitutePanama CityPanama; ^3^ Centre for Ecology & Conservation, College of Life & Environmental SciencesUniversity of ExeterPenrynU.K.

**Keywords:** Aposematism, artificial models, Heliconius, memory, predation

## Abstract

1. Birds are considered to be the primary selective agents for warning colouration in butterflies, and select for aposematic mimicry by learning to avoid brightly coloured prey after unpleasant experiences. It has long been thought that bright colouration plays an important role in promoting the avoidance of distasteful prey by birds.

2. The hypothesis that warning colouration facilitates memorability and promotes predator avoidance was tested by means of a field experiment using distasteful model butterflies. Artificial butterflies with a Heliconius colour pattern unknown to local birds were generated using bird vision models, either coloured or achromatic, and hung in tree branches in a tropical forest. Two sequential trials were conducted at each site to test avoidance by naïve and experienced predators.

3. There was a significant reduction in predation in the second trial. Also, coloured models were attacked less than achromatic models. Specifically, coloured butterflies were attacked significantly less in the second trial, but there was no significant decrease in predation on achromatic models.

4. The present results imply an important role for colour in enhancing aversion of aposematic butterflies. It has also been demonstrated that previous experience of distasteful prey can lead to enhanced avoidance in subsequent trials, supporting mimicry theory.

## Introduction

The aposematic signals of unpalatable prey are a defence against visually hunting predators. In particular, conspicuous colouration is strongly favoured in defended prey as it can increase detection efficiency and lead to rapid decision‐making (Endler, 1988). Colours such as red, yellow, and orange are normally highly contrasting with the background and are commonly used to advertise unpalatability (Stevens & Ruxton, 2012; Arenas *et al.*, 2014). Therefore, these brightly coloured signals support rapid discrimination from cryptic prey and have long been considered to facilitate avoidance learning when compared with less visible colouration (Guilford & Dawkins, 1991; Speed, 2000).

Birds are widely considered to be the primary selective agent for the aposematic colouration of butterflies. After unpleasant experiences with an unpalatable prey, bird predators learn to avoid similar morphs (Ham *et al.*, 2006; Lindström *et al.*, 2006). This learning ability leads to selection favouring the most abundant colour patterns in a local area and generates aposematism and Müllerian mimicry in which predator attacks are reduced through aversion learning of locally common aposematic patterns (Müller, 1879; Mallet & Joron, 1999).

Learning and forgetting are essential for the maintenance of Müllerian mimicry (Speed & Turner, 1999). Memory is linked to recognition, and if predators forget about experiences with prey, then recognition of an aposematic signal is not possible (Speed, 2000). Warning signals should, therefore, be selected to be memorable, to provoke low rates of forgetting and enhance predator aversion (Servedio, 2000; Speed, 2000). Among mimetic butterflies, long‐term memorability of learned avoidance of the model is vital for the protection of the co‐mimic. There is a large body of evidence supporting the role of colour in avoidance learning and memory, but this primarily comes from cage experiments (Sillén‐Tullberg, 1981; Osorio *et al.*, 1999; Ham *et al.*, 2006; Sandre *et al.*, 2010).

Experiments in the wild with natural predators can better estimate the overall response of a local population and can complement cage studies. Responses from captive birds might be influenced by their appetite (Sandre *et al.*, 2010), food deprivation, and artificial environments with constrained viewing, whereas natural environments are heterogeneous and offer a wider variety of alternative food, which might alter decision‐making strategies. For example, a study with natural bird populations using artificial models of the wood tiger moth [*Parasemia plantaginis* (Linnaeus)] suggested that spatial heterogeneity in a predator community creates a mosaic of selection facilitating polymorphism (Nokelainen *et al.*, 2013). Also, another study with wild birds showed that achromatic (non‐coloured) *Heliconius* models were attacked significantly more than coloured models of a local pattern, demonstrating the importance of aposematic signals in avoiding predation (Finkbeiner *et al.*, 2014). Furthermore, an experiment with model poison frogs [*Dendrobates tinctorius* (Schneider)] showed varying attack rates of wild tropical predators in different light conditions (Rojas *et al.*, 2014). Still, few studies, to date, have explored attack rates on different coloured models using wild birds and under natural conditions.

Neotropical *Heliconius* butterflies are one of the best‐studied mimicry systems (Mallet & Joron, 1999), in which unpalatable sympatric species form mimicry rings. Many *Heliconius* species are highly variable in colouration and patterns (Mallet & Gilbert, 1995). Several studies have investigated predator behaviour towards *Heliconius* butterflies in cages using wild‐caught rufous‐tailed jacamars (*Galbula ruficauda* Cuvier), which are specialist predators of fast‐flying insects and exhibit specific butterfly handling strategies. Jacamars readily reject *Heliconius* by sight or by taste and discriminate them from other butterfly species (Chai, 1986; Langham, 2004). Field experiments, using other butterfly predators, kingbirds, and flycatchers, also showed taste‐rejection of *Heliconius* butterflies (Pinheiro, 2003, 2011). Previous field studies have demonstrated mimicry selection by releasing live butterflies (Benson, 1972; Mallet & Barton, 1989) and monitoring recapture rates.

Therefore, to better understand the dynamics of *Heliconius* mimicry, more information from the predators' perspective in the wild is required. Here we investigate the role of colouration in attack rates, testing the ability of bird predators to avoid an unpalatable *Heliconius* warning signal in a tropical forest. The assumption is that wild birds would have a bias against aposematic colouration, which would facilitate the memory of novel butterfly colour pattern. We performed a field test of the hypothesis that aposematism facilitates avoidance of novel distasteful prey using artificial distasteful butterflies with a colour pattern unknown to local bird predators.

## Material and methods

### Production of artificial butterflies

**Figure 1:**
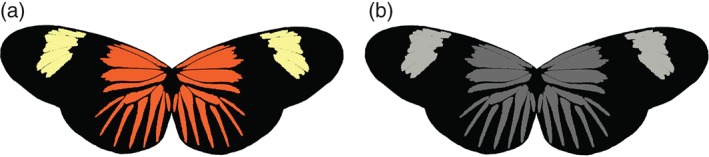
Artificial butterfly models used in the experiment: chromatic (a) and achromatic (b).

Artificial butterflies were produced based on wings of *Heliconius erato lativitta* (Linnaeus) which is found only in the Amazon basin, not in Panama (Brown, 1979; Hines *et al.*, 2011). We calibrated the appearance of the artificial wings to account for bird colour and luminance vision. Photographs of real wings and a printer colour palette were taken with a Fuji‐calibrated UV SLR camera (Fujifilm, Düsseldorf, Germany) with an ultraviolet (UV)‐transmitting quartz lens (Jenoptic) with a UV pass filter (transmitting between 300 and 400 nm; Baader U filter, David Hinds Ltd., Bedfordshire, U.K) and a UV/IR‐Cut pass filter (blocking UV below 400 nm and IR above 700 nm; Baader UV/IR Cut Filter, David Hinds Ltd.), representing the UV and human visible spectrum, respectively. After this, predicted photon catch values of four single cones (used in colour vision) and double cones (probably used in achromatic vision) were calculated, based on the sensitivity of a UV vision bird receptors, Blue tit [*Cyanistes caeruleus* (Linnaeus)] (Hart *et al.*, 2000; Endler & Mielke, 2005), according to the methodology created by Troscianko and Stevens (2015). Our criteria for selecting appropriate colours were that the ‘just‐noticeable‐differences’ (JND) values (Vorobyev & Osorio, 1998) of the printer colours against real butterfly colours (Finkbeiner *et al.*, 2012; Merrill *et al.*, 2012) should be as close as possible to the threshold of discrimination of three JND (Siddiqi *et al.*, 2004) (Table S1 in Supporting information). For achromatic models, only achromatic contrast was used. Colours were closely reproduced as demonstrated in avian colour space vision (Figure S1 in Supporting information). Afterwards, two types of artificial butterflies were designed, coloured, and achromatic (Fig. 1). These were printed on Whatman filter paper (GE Healthcare Life Sciences, Buckinghamshire, U.K.), which produces reflectance spectra close in brightness to actual wings (Finkbeiner *et al.*, 2012), using an HP Colour Laser Jet 4700dn printer (HP Inc., London, U.K.). A 3‐hydroxy‐DL‐kynurenine (3‐OHK, Sigma‐Aldrich Company Ltd., Dorset, U.K.) pigment was applied to the yellow bands of the forewing to provide accurate UV reflectance (Finkbeiner *et al.*, 2012).

The artificial wings were attached with a nylon line to an edible pastry body (flour, lard, water, and black food dye). To provide an unpleasant taste, quinine monohydrochloride dihydrate (4% solution) was sprayed on the body and wings of both model types. This concentration is aversive and has a similar effect to sampling a toxic prey (Rowe & Skelhorn, 2005). Finally, Krylon matte finishing spray was applied lightly to coat the artificial butterflies with a waterproofing element 24 h before placing the models out, without altering the colour or smell.

### Field experiment

The trials were conducted along three forest trails in Parque Nacional Soberanía, Panama. Models were hung by nylon line (∼10 cm long) on tree branches (∼1.70 m high) to swing freely similar to a live butterfly. We aimed to maximise attack rates by butterfly predators that catch insects during flight and detect movement. Models were hung every 10 m in pairs, one coloured and one achromatic on opposite sides of the trail, with the assignment, randomised.

To test memorability, the experiment had two trials. In the first trial, 152 models of each type were placed for 4 days, followed by a second identical trial started 5 days after the first trial finished. In the second trial, the same procedure was repeated at the same location with new 152 new models of each type. The models were checked for attack marks after 48 and 96 h. An artificial model was considered attacked if the body or wings included clearly visible beak marks, or part or all of the body was missing. If a model had more than one beak mark on it, this was counted as a single attack. Evidence of attack by animals other than birds, notably insects such as ants, was readily distinguished and was not counted as an attack (Salazar Carrión et al., 2014).

### Statistical analyses

We used the binomial response of attack (presence or absence) of two treatments (chromatic and achromatic) in two trials (1 and 2) across three localities. To test the homogeneity of variance between localities, a Bartlett test and Fligner–Killeen test were used. We used generalised linear mixed models (GLMM) with a binomial distribution, to test for the effect of trial, treatment, and locality (as a random factor), as well as their interaction terms, on predation. Tests used the R packages stats and lme4 in R statistical software (Bates et al., 2015; R Core Team, 2015).

## Results

**Figure 2:**
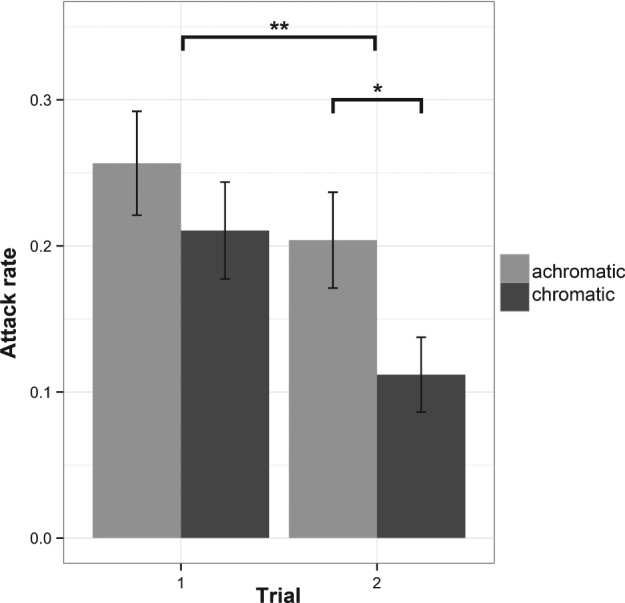
Attack rates on chromatic and achromatic models in sequential trials (±SE) during the first and second trial. Asterisks represent statistically significant P‐values from generalised linear mixed models (GLMM) comparisons, where * between colours, P = 0.031 and ** between trials, P = 0.018.

In total, 608 artificial butterflies were placed in the wild (152 chromatic and 152 achromatic on trial 1 and 152 chromatic and 152 achromatic on trial 2). The use of a nylon line allowed us to recover fully the models, 117 (19%) of which were attacked. Tests of homogeneity revealed no evidence that the three localities differed in predation events (Bartlett test: K^2^ = 0.85, d.f. = 2, P = 0.651, Fligner–Killeen test: χ^2^ = 2.71, d.f. = 2, P = 0.257). The ‘locality’ term did not explain much variation in our model (s^2^ = 0.033, SD = 0.18). There were clear differences in the number of predation events in the models between the two trials (Fig. 2). We observed no difference in predation of the achromatic butterfly between the two trials (37 on trial 1 and 31 on trial 2). A greater proportion of attacks occurred during the first trial (69 on trial 1 and 48 on trial 2, trial: z_604, 608_ = −2.35, P = 0.018). Also, aposematic colour models were attacked less overall (colour: z_604, 608_ = −2.15, P = 0.031). This was mainly as a result of a reduction in attacks in the second trial (32 on trial 1 and 17 on trial 2), but also compared with the achromatic pattern of the second trial (31 achromatic and 17 chromatic). However, although the GLMM showed a significant effect of both trial and colour alone, the interaction between trial and colour was not significant (trial*colour: z_604, 608_ = −1.06, P = 0.28).

## Discussion

We evaluated the influence of aposematic colouration on attack rate by bird predators in a tropical forest. We observed a reduction in attack rates on coloured models as compared to achromatic models, demonstrating a role for colour in enhancing the avoidance of a novel distasteful prey. Many previous experiments have demonstrated the protective value of *Heliconius* warning colour patterns alone (Benson, 1972; Chai, 1986; Mallet & Barton, 1989; Kapan, 2001; Langham, 2004; Merrill *et al.*, 2012; Finkbeiner *et al.*, 2014), including one study which compared chromatic and achromatic prey (Finkbeiner *et al.*, 2014). Our results, therefore, support previous work showing that bright colours enhance the avoidance of aposematic prey, and contributes to an explanation of why aposematic insects in general and *Heliconius* in particular, often evolve bright colouration.

There was a significantly reduced attack rate in the second trial, suggesting that the bad experience of the distasteful model in the first trial may have induced later aversion. Prey palatability is known to influence predator learning and memory of warning colours (Lindström *et al.*, 2006; Skelhorn & Rowe, 2006; Svádová *et al.*, 2009). Having both warning colouration and distastefulness can change predator decision‐making and increase avoidance (Servedio, 2000). However, the short period between trials means that we cannot distinguish between true ‘memory’ and a short‐term aversion reaction to explain these results. It would be interesting to repeat similar experiments over different periods of time to test for long‐term memory. Predation field studies in tropical forests are challenging, and it was not possible to identify predators to demonstrate that the same individual that had a bad experience later avoided the same prey type, so there may be other ecological explanations for our results. Nonetheless, whatever the cause, our experiment supports the prediction of mimicry theory that attack rates on aposematic prey should decline with predator experience.

The avoidance of aposematic patterns is often considered not only as a learned trait but also as an innate response to conspicuous colours, whereby predators are unwilling to eat prey with a novel appearance (Marples *et al.*, 1998; Lee *et al.*, 2010). In addition, a study comparing predation rates on aposematic and cryptic prey, also in field conditions, showed that aposematic prey were completely consumed less often than cryptic prey but partially consumed more often suggesting ‘go‐slow’ predation, in which predators are more cautious with aposematic prey (Carroll & Sherratt, 2013). However, there was no strong support for this in our data, with the two novel patterns equally attacked in the first trial of the experiment. Similar results were found for another *Heliconius* predation experiment in which the ‘nonlocal’ phenotype had higher attack rates (Finkbeiner *et al.*, 2014). Different predators are likely to have different aversion responses to colour, and so the heterogeneity of predators in the wild might explain this result (Endler, 1988; Servedio, 2000; Speed *et al.*, 2000; Endler & Mappes, 2004).

The least attacked prey were the coloured models in the second trial. This suggests that chromatic prey would have triggered a stronger aversion response than the achromatic prey, implying a role for colour in reducing attack rates. However, a test for the interaction between trial and pattern was not significant, so we cannot definitively conclude that colour influenced the reduced response in the second trial, although this seems likely. A power analysis suggested that we would need to quadruple the size of our experiment approximately to detect a significant interaction between colour and trial. The results are nonetheless consistent with the idea that colour enhances learning of aversion (Speed, 2000).

Predator psychology models assume that the rate of predation is dependent on learning and forgetting rates, and the absence of reinforcing experiences might lead to forgetfulness (Turner & Speed, 1996; Speed & Turner, 1999; Servedio, 2000; Speed, 2000). For instance, Jacamars have been shown to forget novel colour morphs after an interval of 2 years (Langham, 2004), which might have been as a result of a lack of reinforcing encounters with the artificial prey. Our artificial butterflies were in the sight of predators for 4 days during the trials, which may have led them to be seen several times and which could have stimulated memory. Occasional sampling in nature also might reinforce memory provided that butterflies can be rejected by sight or by taste, which is a common behaviour among butterfly predators (Chai, 1986; Pinheiro, 2003). Further experiments would be needed to determine whether distasteful models or repeated exposure could trigger long‐term memory and faster learning rates.

In this experiment, there were no detectable effects of the pattern itself as a warning signal, as the distasteful achromatic pattern was equally attacked in both trials. Previous experiments with chicks indicate that colour differences are more memorable than luminance contrast, whereas pattern attracts attention (Osorio *et al.*, 1999). Nonetheless, previous studies have shown avoidance learning using different patterns (Rowe *et al.*, 2004; Aronsson & Gamberale‐Stille, 2008; Rowland *et al.*, 2010), and benefits of pattern mimicry may emerge at a later stage in the learning process (Rowe *et al.*, 2004). Given the precise mimicry is seen in *Heliconius*, both pattern and colour seem to be vitally important for predator avoidance (Finkbeiner *et al.*, 2014).

The attack frequency of this study was significantly higher than in previous work using artificial *Heliconius* patterns (Merrill *et al.*, 2012; Finkbeiner *et al.*, 2014; Salazar Carrión *et al.*, 2014). This may be partly because the models represented a novel morph that birds had not experienced before. However, our methodology using suspended butterflies that could move in the wind might also have attracted more predators. This method may, therefore, be useful for future experiments studying selection on butterfly models.

This experiment indicates that attack rates on novel aposematic butterflies are reduced over time, consistent with experiments on caged birds showing learning of warning colours. Furthermore, we have also shown a role for colour in enhancing aversion towards aposematic prey. This experiment has shown avoidance of an aposematic butterfly in a tropical forest and contributes to a better understanding of the dynamics of *Heliconius* aposematic mimicry in the wild.

## Supporting information


**Table S1.** Chromatic and achromatic contrast (JND) between the real wing and printed wing perceived by Blue tit (Cyanistes caeruleus) vision. Values >3 JND denote an increasing ability of discrimination, whereas values ≤3 JND denote colours generally indistinguishable from each other. Notice that yellow colour could be closely reproduced in the models and the orange was close but not possible to reproduce accurately.
**Figure S1.** Distribution of colours perceived by Bluetit (Cyanistes caeruleus) vision in a tetrahedral colour space. Each point is determined by the relative stimulation of the four cone colour channels and each axis represents a channel: ultraviolet (UV), short (SW), medium (MW), and long (LW) wavelength sensitive cones.
