# Supplementary material for: Avoidance of an aposematically coloured butterfly by wild birds in a tropical forest
Source: Ecol Entomol. 2016 Jun 25;41(5):627–32. doi: 10.1111/een.12335 (PMC5026159; doi:10.1111/een.12335)

Supporting Information for

Avoidance of an aposematically coloured butterfly by wild birds in a tropical forest

Table S1. Chromatic and achromatic contrast (JND) between real wing and printed wing perceived by Blue tit (*Cyanistes caeruleus*) vision. Values > 3 JND denote an increasing ability of discrimination, whereas values ≤ 3 JND denote colours generally indistinguishable from each other. Notice that yellow colour could be closely reproduced in the models and the orange was close but not possible to reproduce accurately.

	JND	
	Chromatic	Achromatic
Orange	7.3	8.7
Yellow	4.9	5.7
Grey Orange	-	7.8
Grey Yellow	-	2.8

Figure S1. Distribution of colours perceived by Bluetit (*Cyanistes caeruleus*) vision in a tetrahedral colour space. Each point is determined by the relative stimulation of the four cone colour channels and each axis represent a channel: ultraviolet (UV), short (SW), medium (MW) and long (LW) wavelength sensitive cones. Notice that yellow colour could be reproduced in the models and the orange was close but not possible to reproduce accurately.

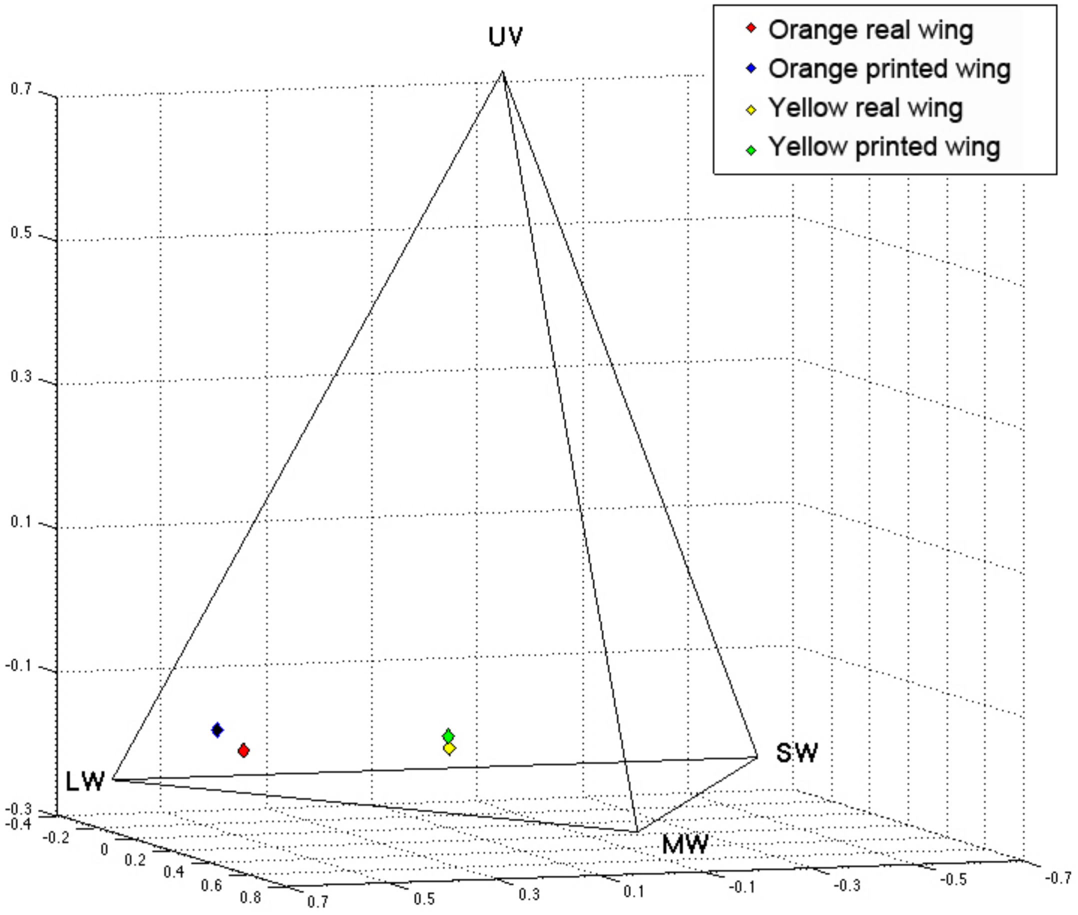

Supplement: Supplementary file 1 — Table S1. Chromatic and achromatic contrast (JND) between the real wing and printed wing perceived by Blue tit (Cyanistes caeruleus) vision. Values >3 JND denote an increasing ability of discrimination, whereas values ≤3 JND denote colours generally indistinguishable from each other. Notice that yellow colour could be closely reproduced in the models and the orange was close but not possible to reproduce accurately. Figure S1. Distribution of colours perceived by Bluetit (Cyanistes caeruleus) vision in a tetrahedral colour space. Each point is determined by the relative stimulation of the four cone colour channels and each axis represents a channel: ultraviolet (UV), short (SW), medium (MW), and long (LW) wavelength sensitive cones. [file EEN-41-627-s001.pdf]
